# The correlation between malaria RDT (Paracheck pf.®) faint test bands and microscopy in the diagnosis of malaria in Malawi

**DOI:** 10.1186/s12879-017-2413-x

**Published:** 2017-05-02

**Authors:** Ryoko Makuuchi, Sandy Jere, Nobuchika Hasejima, Thoms Chigeda, January Gausi

**Affiliations:** 1 Nippon International Cooperation for Community Development, 101 Nishi-rokkaku-cho, Nakagyo-ku Kyoto, 604-8217 Japan; 2 Ministry of health, Community Health Science Unit, PO Box 30377, Lilongwe, Malawi; 3 Lilongwe District Health Office, PO Box 1274, Lilongwe, Malawi

**Keywords:** Malaria rapid diagnostic test, Paracheck pf.®, Faint test band, Sensitivity, Specificity, PPV, NPV, Accuracy of diagnosis

## Abstract

**Background:**

Faint test bands of Paracheck Pf.® are interpreted as malaria positive according to world health organization (WHO) guideline. However if there are conspicuous number of faint test bands, a performance of Paracheck Pf.® could be influenced depending on whether interpreting faint test bands as malaria positive or negative. Finding out the frequency and accurate interpretation of faint test bands are important to prevent the overdiagnosis and drug resistance.

**Methods:**

A cross-sectional, descriptive study was conducted to find out the frequency of faint test bands and evaluate the performance of Paracheck Pf.® by sensitivity, specificity, positive predictive value (PPV), negative predictive value (NPV) and accuracy of diagnosis of Paracheck Pf.® using microscopy as the gold standard. 388 suspected patients with malaria in Malawi were recruited in this study. Malaria rapid diagnostic tests (RDTs) and microscopy were used and patients’ information which includes age, sex, body temperature and signs or symptoms of malaria were recorded.

**Results:**

Among all patients involved in the study, 29.1% (113/388) were found malaria positive by RDT. Overall 5.4% (21/388) of all Paracheck Pf.® tests resulted in a “faint test band” and 85.7% (18/21) corresponded with malaria negative by microscopy. Faint test bands which corresponded with malaria positive by microscopy were lower parasite density and there are no patients who showed definitive symptom of malaria, such as fever. When Paracheck Pf.® “faint test bands” were classified as positive, accuracy of diagnosis was 76.5% (95% CI 72%–80.7%) as compared to 80.4% (95% CI 76.1%–84.2%) when Paracheck Pf.® “faint test bands” were classified as negative.

**Conclusions:**

This study shows that frequency of faint test bands is 5.4% in all malaria RDTs. The accuracy of diagnosis was improved when faint test bands were interpreted as malaria negative. However information and data obtained in this study may not be enough and more intensive research including a frequency and property of faint test bands is needed for significant interpretation of faint test bands.

## Background

Malaria is a major public health problem in Malawi with at least six million cases occurring annually [1]. Early and accurate diagnosis is important in an effective management of malaria. According to the WHO guideline, it recommends confirmation of the diagnosis of malaria using either malaria rapid diagnostic tests (RDTs) or microscopy for all suspected cases before the administration of treatments [2, 3]. Malawi also follows this WHO guideline and sets up the strategic plans including expansion of microscopic diagnosis of malaria parasite in central and district hospitals as well as in facilities with high patient loads [4]. However, due to the lack of laboratory equipment and trained staffs, using malaria RDTs is very common in peripheral health facilities. One study (Allen, 2011) focusing on a faint test band of Paracheck Pf. ® mentions the possibility of an over diagnosis showing the data that 23.7% of all participants were faint test bands and 94.2% of them were negative by microscopy [5].

Nippon International Cooperation for Community Development (NICCO) is one of the Japanese NGOs which was conducting medical projects including malaria control, schistosomiasis control, maternal and child health activities, mobile clinics and the bicycle ambulance program in Lilongwe district, Malawi. Malaria RDTs were provided in the malaria control and the mobile clinic. While providing RDTs through those activities, it was found that some malaria RDT results were showing faint test bands with lines on the malaria RDT cassette appearing not visible enough and requiring good light to be seen. According to the WHO guideline, faint test bands of the malaria RDT are interpreted as malaria positive [6]. The Ministry of Health of Malawi follows the same guideline [7].

There are many studies reported about a performance of Paracheck Pf.® or malaria RDTs based on the detection of histidine-rich protein 2(HRP-2). However the topic of the faint test band frequency was referred only in the study by Allen et al. that was conducted in low transmission area of Tanzania [5]. The study reported that among 291 participants who were tested by Paracheck Pf.®, 74.6% (217/291) participants were negative, 1.7% (5/291) were positives and 23.7% (69/291) were faint test bands. If faint test bands are interpreted as malaria positive, the percentage of faint test bands was 93.2% (69/74) in the malaria positive. Among those faint test band cases, 94.2% (65/69) corresponded with malaria negative by microscopy.

If there are conspicuous number of faint test bands, accuracy of Paracheck Pf.® could be changed depending on how to interpret faint test bands, whether interpreting them as positive or negative. However the information about faint test bands such as its frequency or its causes are limited. This study was conducted to examine the frequency of faint test bands and the accuracy of Paracheck Pf.® by using microscopy as the gold standard.

## Methods

### Study site

Malawi is divided into 28 districts and each district is divided into several areas which are called Traditional Authority (T/A) [8, 9]. NICCO’s medical project was conducted in one of the T/As called Malili in Lilongwe district since December 2013. In T/A Malili, there were 997 households and a population of 4494 people (20% of this population were children under age five). It takes 12 km to reach nearest health center and about 30 km to the district hospital of Lilongwe city. As one of the activities of NICCO’s medical project, the mobile clinic was operated in this area for the purpose of providing health cares for people who are living far from medical facilities. Provided health cares were included measuring blood pressure and body temperature, urine dip stick test, malaria RDT, HIV counseling and testing, prenatal checkup, consultation by clinicians and prescription. People who came to this mobile clinic were mostly diagnosed by malaria, respiratory infections, diarrhea and hypertension. Malaria is most common disease in this area, especially in a rainy season. Four malaria mass check-ups were conducted in this area during two years for the purpose of understanding the periodical prevalence of this area in both rainy season and dry season (February 2013, November 2013, April 2014 and November 2014). From the statistics of these mass check-ups, the rate of participants who showed positive results by the malaria RDT (Paracheck Pf.®) in the rainy seasons and the dry seasons were approximately 70% and 30% respectively.

This study was conducted during the mobile clinic in T/A Malili targeting the patients who were suspected malaria.

### Study design

This is a cross sectional, descriptive study which was carried out in June to November in 2014 in Lilongwe, Malawi. Among 747 patients who came to NICCO’s mobile clinic during this period, 406 (54%) patients showed and/or complained signs and symptoms of malaria. Among them 388 (96%) patients (female 241, male 147, under five years children 151, maximum age 94 years old, minimum age 3 months) were involved in the study. Malaria RDTs using Paracheck Pf. ® were done to all patients who were suspected malaria. The results which include positive, negative and faint test band were recorded and sensitivity, specificity, negative predictive value (NPV), positive predictive value (PPV) and accuracy of RDT were evaluated using microscopy as the gold standard. Participants’ information which includes age, sex, body temperature and signs or symptoms of malaria were recorded.

### Exclusion criteria

Patients who received an anti-malarial treatment within last 14 days and patients who did not agree to participate in the study were not included.

### Paracheck pf.®

Paracheck Pf.® (Orchid Biomedical Systems, Verna Goa, India) was used to diagnose malaria. The choice of Paracheck Pf.® was based on its low cost, high sensitivity (98.6%) in its controlled trials, simple to use by health surveillance assistants and was recommended by the Ministry of Health [7]. The test kits were stored in one of the rooms in NICCO’s office which was cool and dry location after purchasing from the pharmacy. These were carried to the mobile clinic site using plastic storage cases. Malaria RDTs using Paracheck Pf.® were performed for malaria suspected patients by trained health surveillance assistants following the standard protocol [3]. One qualified lab technologist from Lilongwe District Laboratory was there for the supervision. Blood taken from the patients’ finger was transferred using the provided sample collecting device to the test window and then two drops of clearing buffer were added to the buffer well. Once a Paracheck Pf.® kit was opened, it was used immediately. Trained health surveillance assistants interpreted RDT results exactly 20 min following the addition of the buffer solution as recommended by the manufacture. In order to ensure the integrity of interpretation, all research personnel involved in the Paracheck Pf.® interpretation were blinded to both the results of microscopy as well as the clinical decision.

### Interpretation of Paracheck pf.®

The appearance of both a control band and a test band was concluded as a positive for *P. falciparum* [7]. The appearance of a control band and the absence of a test band concluded as a negative. Faint test bands were those bands that were only visible in a good light [6].

### Microscopy

In the study site, blood was taken from finger prick and thick and thin smears were made by two skilled lab technologists of Lilongwe District Health Office Laboratory under the Ministry of Health following the guideline of the WHO [10]. Those lab technologists have a diploma in biomedical sciences and had more than three years of experience. Slides were brought to the laboratory of Community Health Sciences Unit (CHSU) which is under the Ministry of Health and responsible for conducting researches regarding tuberculosis, HIV, maternal and child health, schistosomiasis and malaria. Two laboratory technologists at the national reference laboratories in the Ministry of health at CHSU worked on staining collected blood film slides with Gimsa stain, parasite speciation and quantification. The technologists performing these tests were blinded to the results of malaria RDT and patients’ clinical information. A third technologist examined all discordant results as a tie breaker and was also blinded to the results of malaria RDT, patients’ clinical information and the results of microscopy by two lab technologists. All technologists who worked on this series of quality control procedure were well qualified with at least 10 years of malaria microscopy.

### Quality control

- Paracheck Pf.®

The procedure of health surveillance assistants on RDTs was always followed by the protocol and supervised by the lab technologists [3]. Faint test bands were observed by at least two health surveillance assistants and considered as a true faint test band only if both of the observers agreed to it.

b) Microscopy

Readings were conducted at CHSU and all procedures were followed by lab technologists as mentioned earlier. Two lab technologists read all samples and recorded the results by the plus system (+: 1–10 per 100 thick fields, ++: 11–100 per 100 thick fields, +++: 1–10 per thick field, ++++: >10 per thick field.) All lab technologists were blinded each other as well as the results of Paracheck Pf.®. Evaluation of those two readers’ interrater reliability was 95.62% agreement (kappa = 0.8872, *P* = 0.0000). There were 17 samples out of 388 that 1st and 2nd readers did not agree. Only those discordant samples were rechecked by the 3rd reader who was the quality control technologist, the tie breaker. The 3rd reader was blinded to the 1st and 2nd readers’ microscopy results and Paracheck Pf.® results.

### Clinical profile of study participants

Among 388 participants, 151 (39%) were children under 5 years old, 70 (18%) were children aged between 6 and 15 years old and 167 (43%) were adult over 16 years old. Fever were seen in 10% of participants and all participants were showed and/or complained other minor signs and symptoms such as cough, feeling feverish, general body pain, headache, abdominal pain, watery diarrhea, and vomit. (Table 1) Even though patients did not have body temperature over 37.5° when they measured at the mobile clinic, they were tested malaria RDTs if clinicians thought the necessities of RDTs during the history taking and face-to-face consultation.

**Table 1:** Participants’ profile

	Number (%) *n* = 388
Male	147 (37.9%)
Female	241 (62.1%)
Age range	
0-5 years	151 (39.0%)
6-15 years	70 (18.0%)
Over 15 years	167 (43.0%)
Body temperature	
37.5° - 38.4°	23 (5.9%)
> 38.5°	17 (4.4%)
Patients’ complaints (multiple answers)	
Cough	175
Feeling feverish	174
General body pain	102
Headache	74
Abdominal pain	64
Watery diarrhea	44
Vomit	24

### Data analysis

All data were entered into Microsoft Excel. The data analysis was done with STATA version 11.2. Data from malaria RDT and microscopy results were tested for homogeneity using the McNemar’s test. Accuracy of Paracheck Pf.® was evaluated by calculating sensitivity, specificity, PPV, NPV and diagnostic accuracy with 95% confidence interval (CI). Sensitivity of Paracheck Pf.® was measured as the proportion of RDT positive over the total positive determined by microscopy. Specificity of Paracheck Pf.® was measured as proportion of RDT negative over the total negative determined by microscopy. The PPV is the number of RDT true positives divided by the number of all RDT positive test results (true positives plus false positives). The NPV is the number of RDT true negatives divided by the number of all RDT negative test results (true negatives plus false negatives). The accuracy of diagnosis is a sum of true positives and true negatives divided by true positives, false positives, true negatives and false negatives.

## Results

Among 388 patients involved in the study, 29.1% (113/388) were RDT positives, 5.4% (21/388) were faint test bands and 65.5% (254/388) were RDT negatives. If faint test bands are interpreted as malaria positive, the percentage of faint test band was 15.7% (21/134) in the malaria positive. Baseline characteristics of patients in RDT positive, negative and faint test band are showing in Table 2.

**Table 2:** Baseline characteristics of patients in RDT positive, negative and faint test band

	RDT positive (%) *n* = 113	RDT negative (%) *n* = 254	Faint test band (%) *n* = 21
Sex			
Male	64 (56.6%)	92 (36.2%)	6 (28.6%)
Female	49 (43.4%)	162 (63.8%)	15 (71.4%)
Age range			
< 5 years	65 (57.5%)	82 (32.3%)	4 (19%)
6–15 years	30 (26.5%)	34 (13.4%)	6 (28.6%)
> 16 years	18 (16.0%)	138 (54.3%)	11 (52.4%)
Fever (>37.5°)	21 (18.6%)	19 (7.5%)	0

From microscopy, 55.8% (63/113) of all RDT positive cases were true positive and 44.2% (50/113) were false positive. Among RDT negative cases, 9.1% (23/254) were false negative and 90.9% (231/254) were true negative. Out of 21 faint test band cases, 14.3% (3/21) were malaria positive and 85.7% (18/21) were malaria negative by microscopy. All microscopic positive cases were identified as *Plasmodium falciparum*.

Those three faint test band cases showing true positive on microscopy were lower parasite density, one plus (+) or two plus (++), by qualitative reading. Among all 21 faint test band cases, there were no patients with a body temperature over 37.5° which is the major symptom of malaria (Table 3).

**Table 3:** Clinical profile of patients who showed a faint test band

	Age range	Sex	Body temperature	Patients’ complaints	Microscopy result
1	>16 years old	F	<37.5	GBP^a^	−
2	>16 years old	F	<37.5	Feeling feverish, cough	−
3	6–15 years	M	<37.5	Feeling feverish, cough	−
4	6–15 years	F	<37.5	GBP, feeling feverish, cough	−
5	6–15 years	F	<37.5	Cough	−
6	>16 years	F	<37.5	Headache, muscle pain	−
7	6–15 years	F	<37.5	Headache	−
8	<5 years	M	<37.5	Feeling feverish, cough	−
9	<5 years	F	<37.5	Feeling feverish, cough	−
10	6–15 years	F	<37.5	Feeling feverish	++
11	<5 years	F	<37.5	Feeling feverish, cough	−
12	>16 years	M	<37.5	GBP, headache	−
13	>16 years	F	<37.5	GBP, headache	−
14	>16 years	F	<37.5	GBP, cough	−
15	<5 years	M	<37.5	Feeling feverish, cough	−
16	>16 years	F	<37.5	GBP, feeling feverish, cough, abdominal pain	−
17	6–15 years	M	<37.5	Feeling feverish, abdominal pain	−
18	>16 years	F	<37.5	GBP, feeling feverish, cough	+
19	>16 years	F	<37.5	GBP, feeling feverish	−
20	>16 years	M	<37.5	GBP, headache	+
21	>16 years	F	<37.5	Feeling feverish, headache	−

^a^GBP = general body pain

**Fig. 1:**
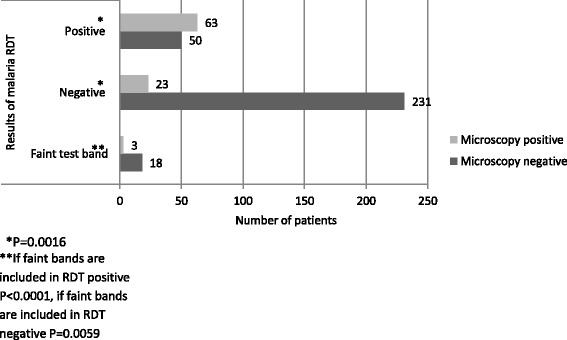
Comparison of Microscopy and malaria RDT (Paracheck Pf.®) results

When faint test bands were interpreted as malaria positive, 50 out of 113 RDT positive cases and 18 out of 21 faint band cases were actually malaria negative by microscopy. It means 50.7% (68/134) of malaria RDT positive cases resulted in false positive. Sensitivity and specificity of the RDT compared with microscopy were 74.2% (CI 63.8% - 82.9%) and 77.3% (CI 72.1% - 81.9%). Positive predictive value (PPV) and negative predictive value (NPV) were 49.3% (CI 40.5% - 58%) and 90.9% (CI 86.7% - 94.2%). The diagnosis of accuracy was 76.5% (CI 72% - 80.7%). (Fig. 1).

When the faint test band was interpreted as RDT negative, sensitivity and specificity of RDT compared with microscopy were 70.8% (CI 60.2% - 79.9%) and 83.3% (CI 78.6% - 87.3%) respectively. PPV and NPV were 55.8% (CI 46.1%–65.1%) and 90.5% (CI 86.5%–93.7%). Accuracy of diagnosis is 80.4% (CI 76.1% - 84.2%). When the faint band was interpreted as RDT negative, the performance of Paracheck Pf. ® changed; sensitivity decreased from 74.2% to 70.8%, specificity increased from 77.3% to 83.3%, PPV increased from 49.3% to 55.8%, NPV decreased from 90.9% to 90.5% and the accuracy of diagnosis increased from 76.5% to 80.4% (Table 4).

**Table 4:** Performance of Paracheck Pf.® between interpreting faint test bands as malaria positive and negative

Interpretation of faint test bands	Sensitivity (number/total) [95%CI]	Specificity (number/total) [95%CI]	PPV (number/total) [95%CI]	NPV (number/total) [95%CI]	Accuracy of diagnosis (number/total) [95%CI]
Faint test bands as malaria (+)	74.2% (66/89) [63.8–82.9]	77.3% (231/299) [72.1–81.9]	49.3% (66/134) [40.5–58]	90.9% (231/254) [86.7–94.2]	76.5% (297/388) [72–80.7]
Faint test bands as malaria (−)	70.8% (63/89) [60.2–79.9]	83.3% (249/299) [78.6–87.3]	55.8% (63/113) [46.1–65.1]	90.5% (249/275) [86.5–93.7]	80.4% (312/388) [76.1–84.2]

## Discussion

Among all patients involved in this study, 5.4% (21/388) of them showed faint test bands. If faint test bands are interpreted as malaria positive, the frequency of faint test band was 15.7% (21/134) in RDT positive. This frequency is not very high like the study by Allen et al. which showed the frequency of faint test band as 23.7% among all patients. [5] We could find other 38 reports about Paracheck Pf.® through Pubmed but none of them reported the frequency of faint test bands except the report by Allen et al. [11–48]. However, Kyabayinze et al. mentioned about the faint test bands of Paracheck Pf.® in their research. The research followed up patients with malaria for 63 days to examine the time course of persistent antigenicity. Among 1557 times of Paracheck Pf.® provided during the follow up period, faint test bands were seen in 29.7% (462/1557) of them [19]. Sinha et al. carried out the research which includes measuring interobserver variability for each of four kinds of RDT brands. The interobserver variability assessment were done for Paracheck Pf. ® and kappa-statistic was recorded depends on the level of band intensity; 0 (no visible band), 1 (faint line), 2 (faint band), 3 (clear band weaker than control) and 4 (clear band equivalent to or more intense than positive control). Though this research did not mention the faint test band frequency itself, it implied that the band of Paracheck Pf. ® was able to be sorted by the level of intensity and faint test bands were recognized by the researchers [20]. McMorrow et al. examined the sensitivity and specificity of Paracheck Pf.® and its change after providing the training on RDT’s performance to health workers. In this report, it is well understood that the authors recognized faint test bands by mentioning that "samples with low-density parasitemia (200-500 parasites/μl) produce faint positives" and "reading the test too soon may prevent a detection of the faint positives because of the continued of hemolyzed blood" [16]. There was one study which did not specify “faint test bands” but “unclear band”. Through the study which evaluated the performance of Paracheck Pf. ® by Proux et al., the result of Paracheck Pf. ® was graded as negative, positive, unclear or invalid. This study also implied that researchers could recognize the band intensity or its visibility [21]. Moreover, Batwala et al. also recognized the existence of faint test bands and defined its interpretation in their study which was done for assessing the accuracy of Paracheck Pf.®. Under the description of laboratory procedure, it was written that faint test bands of RDT were considered as positive and the third reader’s result was final if the readings of faint test bands were discrepant [11].

Several other studies used different kinds of malaria RDT brands apart from Paracheck Pf. ® were also found. Palen et al. examined the correlation between the line intensity of RDTs and parasite densities using the RDT brands of SD FK50 Malaria Ag *P.falciparum* and SD FK60 Malaria Ag *P.falciparum*/Pan. Faint or weak line intensities occurred in 32.1% (98/305) of true positive results of SD FK50 Malaria Ag *P.falciparum*It, and in 29.5% of true positive results of SD FK60 Malaria Ag *P.falciparum* / Pan. It explained that those faint or weak line intensities were occurred mostly but not exclusively at low parasite densities [49]. This study was expanded to further study by Gillet et al. which examined the correlation between RDT line intensities and parasite densities among the samples specifically showing the prozone effect. The several RDT brands were used and a band intensity was divided into four levels; none (no line visible), faint (barely visible line), weak (paler than control line), medium (equal to the control line) and strong (stronger than the control line). The Results showed that among 51 samples with the prozone effect, four (7.8%) showed faint bands and three (5.9%) resulted in faint or weak bands depending on the observer [50].

As mentioned previously, totally eight studies recognized the existence of faint test bands and/or reported the faint test band frequency of Paracheck Pf.® or other RDT brands [5, 11, 16, 19–21, 49, 50]. The other studies regarding Paracheck Pf.® mentioned only malaria positive or negative and did not mention faint test bands [12–15, 17, 18, 22–48]. The reasons of this paucity of studies mentioning the frequency of faint test band could be that the number of faint test bands was very small or faint test bands were not paid much attention. We evaluated a performance of Paracheck Pf.® in two different groups, one is the interpretation that faint test bands are considered as malaria positive and the other is negative. As a result, if those 21 faint test bands are interpreted as malaria positive, sensitivity, specificity, PPV and NPV were 74.2%, 77.3%, 49.3% and 90.9%. [Table 4] If faint test bands are interpreted as malaria negative, sensitivity decreases about 3%, specificity and PPV increased about 6%, and NPV stayed about the same. And the accuracy of diagnosis is higher (80.4% CI 76.1%–84.2%) when faint test bands were interpreted as malaria negative rather than as positive (76.5% CI 72%–80.7%). [Table 4] From this data the interpretation of faint test band influenced slightly on an accuracy of Paracheck Pf.®. For improving the accuracy of the test, faint test bands cases are better to be interpreted as malaria negative only if they are not vulnerable individuals such as children under five years, pregnant women or immune-compromised. This will help to prevent overdiagnoses, prevent an emergence of drug resistant malaria and save variable resources.

From our intensive literature search, the sensitivity of Paracheck Pf.® varies from 54% in Nigeria by Flade CO et al. [14] to 100% in Congo by Swarthout et al. [15]. Specificity varies from 52% in Congo by Swarthout et al. [15] to 100% in Vietnam by Huong et al. [47]. The sensitivity and specificity of our result were low compared to previous researches. The reason for this low sensitivity and specificity are not clear but various factors which decrease the accuracy of RDT were reported [5, 16, 50]. The factors for reducing sensitivity are deletions or mutations in the Pf HRP-2 gene, the prozone effect, and the inappropriate using of the loop blood transfer device attached with a RDT kit [5, 50]. The factors for reducing specificity are prolonged HRP2 after eliminating parasites, and using a qualitative reading for parasitemia [16].

When considering the possible factors of faint test band appearance, parasite density could be also one of them. The WHO guideline mentions that the lines can be faint at low parasitemia [51]. Our study shows that out of 21 faint test band cases none had a body temperature over 37.5°, this means none of them had the main symptom of malaria. Also, among those 21 faint test band cases 85.7% (18/21) were malaria negative and only three were malaria positive by microscopy. A qualitative reading for parasitemia (e.g. +,++,+++,++++) was used and it found that one out of three malaria positive cases was two plus (++), the others were one plus (+) which are the lower level of parasitemia. These results infer that lower parasitemia may tend to form a false negative as WHO mentioned.

From this consequence, it can be said that the patients who show faint test bands should be treated as malaria positive in areas where medical circumstances are limited such as our research area.

The information and data obtained in this study were not enough to conclude an accurate interpretation of faint test band since there are limitations of the study such as a small number of faint test band samples, not conducting quantitative reading for blood smears, not using PCR and not observing the patients chronologically. Further studies measuring frequency of faint test band and defining its interpretation are greatly needed. It is because that clinical diagnosis and treatment will be influenced by faint test bands especially in local health facilities of a high malaria transmission area where the malaria RDT is the most reliable and only available examination.

## Conclusion

If faint test bands are interpreted as malaria positive, the frequency of faint test bands was 15.7% in the RDT positive. The result of sensitivity, specificity, PPV and NPV of Paracheck Pf.® could not make a conclusion of an ideal interpretation of a faint test band. However the accuracy of diagnosis when faint test bands were interpreted as malaria negative is higher than when these were interpreted as positive. Furthermore, according to the result of microscopy as the gold standard, over 85% of faint test bands were malaria negative. From these results, it is possible to interpret faint test bands as malaria negative. This can be applied only if patients do not have risk factors of getting severe malaria such as age below five years, pregnancy, travelers from non-malaria epidemic areas or immune deficiency. Considering the data obtained in this study and the possibility that an inadequate interpretation of faint test band may influence on the patients’ diagnosis and proper treatment, more intensive research focusing on faint test bands, its frequency and evaluating an accurate interpretation of faint test bands using PCR as the gold standard are highly recommended.

## Abbreviations

CHSU: Community Health Science Unit
CI: Confidence Interval.
HRP-2: histidine-rich protein 2(HRP-2).
NHSRC: National Health Sciences Research Committee.
NICCO: Nippon International Cooperation for Community Development.
NPV: Negative Predictive Value.
PCR: Polymerase Chain Reaction.
PPV: Positive Predictive Value.
RDT: Rapid Diagnostic Test.
T/A: Traditional Authority.
WHO: World Health Organization.
